# Defining end of life in dementia: A systematic review

**DOI:** 10.1177/02692163211025457

**Published:** 2021-06-17

**Authors:** Bria Browne, Nuriye Kupeli, Kirsten J Moore, Elizabeth L Sampson, Nathan Davies

**Affiliations:** 1 Centre for Ageing Population Studies, Research Department of Primary Care and Population Health, University College London, London, UK; 2 Marie Curie Palliative Care Research Department, University College London, London, UK; 3 National Ageing Research Institute, Parkville, Victoria, Australia; 4 Barnet, Enfield and Haringey Mental Health Trust, North Middlesex University Hospital, London, UK

**Keywords:** Dementia, end of life, terminal care, palliative care, systematic review

## Abstract

**Background:**

Dementia is a life-limiting condition that affects 50 million people globally. Existing definitions of end of life do not account for the uncertain trajectory of dementia. People living with dementia may live in the advanced stage for several years, or even die before they reach the advanced stage of dementia.

**Aim:**

To identify how end of life in people with dementia is measured and conceptualised, and to identify the factors that contribute towards identifying end of life in people with dementia.

**Design:**

Systematic review and narrative synthesis.

**Data Sources:**

Electronic databases MEDLINE, EMBASE, PsychInfo and CINAHL, were searched in April 2020. Eligible studies included adults with any dementia diagnosis, family carers and healthcare professionals caring for people with dementia and a definition for end of life in dementia.

**Results:**

Thirty-three studies met the inclusion criteria. Various cut-off scores from validated tools, estimated prognoses and descriptive definitions were used to define end of life. Most studies used single measure tools which focused on cognition or function. There was no pattern across care settings in how end of life was defined. Healthcare professionals and family carers had difficulty recognising when people with dementia were approaching the end of life.

**Conclusion:**

End-of-life care and research that focuses only on cognitive and functional decline may fail to recognise the complexities and unmet needs relevant to dementia and end of life. Research and clinical practice should adopt a needs-based approach for people with dementia and not define end of life by stage of disease.

**What is already known about the topic?**
  - General end of life definitions cannot be applied to people with dementia, as the advanced stage of dementia may persist for several years and be very unpredictable.
  - People with dementia are less likely to receive palliative care, due to the complex and unpredictable disease trajectory. This can result in unmet needs and potentially burdensome interventions until death.
**What this paper adds?**
  - Inconsistent methods are used to define end of life in dementia research and practice. Most studies rely on a single validated tool that assesses cognition or ambulatory function. Tools and policies that rely on a chronological progression of stages may not reflect the variability in how dementia progresses.
  - Most studies focus on functional or cognitive measurements of end of life and do not consider the holistic needs of the individual.
**Implications for practice, theory or policy**
  - Future research and clinical care need to avoid single domain measures to define end of life in dementia.
  - There is a need to refocus discussion to a needs-based approach to care which adopts a palliative approach tailored for people living with dementia that encompasses physical, medical and psychosocial needs.
  - There is a need for a clear consensus on what defines ‘end of life’ in dementia, to inform policies and practices and promote adoption of a needs-based approach, to allow appropriate care in a timely manner.

## Introduction

Dementia currently affects over 50 million people globally, and this is estimated to increase to 152 million by 2050.^1,2^ Although dementia mainly affects people over the age of 60, this condition is not a normal part of ageing.^3^ Therefore, dementia is considered a global public health priority. According to the latest World Health Organization’s Global Burden of Disease report, dementia is the fourth main cause of disability among people aged 75 and older.^4^ The independent contribution of dementia to mortality is currently difficult to assess, as older people commonly have co-morbidities that may or may not be related to dementia, which can also shorten their lives.^3^ Although dementia is a known progressive and neurodegenerative disease,^5^ it may not be considered life limiting in clinical practice, which results in a lack of specialised care for people with dementia approaching the end of life, and deaths not being attributed to dementia.^3,6^

A variety of definitions of end of life have evolved over time.^7^ The Department of Health in the United Kingdom (UK) defines end of life as the period when a person with an advanced, progressive or incurable condition may die within 12 months.^8^ Understanding the timing of when a person is actively dying is important at the individual level, where the person and their family can make preparations for the end of life. This is also important at a policy level, where interventions involving end of life care can be prioritised.^9^ General definitions of end of life may not be feasible to apply to dementia, as people with advancing dementia may continue to live for several years.^10^

The dementia trajectory is variable with progressive decline, punctuated by acute events such as an infection or falls, where the person may recover or experience an increased rate of decline in health until the end of life.^11^ This is the point where a dementia prognosis displays unpredictability, and can vary between and within individuals.^12^ In the advanced stages, people with dementia experience potentially burdensome interventions near the end of life, as their physical, spiritual and psychosocial needs are not addressed in a timely manner.^13^

Palliative care is a multidisciplinary approach that improves the quality of life for patients and their families, who encounter challenges associated with life-limiting conditions.^14^ This is achieved by performing early assessments and identification of physical, psychosocial and spiritual needs. End of life care refers to the care for people with a disease once they have reached a rapid decline in health.^15^ The period of when people are considered to be at these stages may vary in regulatory or policy guidelines in different areas of the world.^16^ The White Paper expert consensus of the European Association of Palliative Care, argues that optimal palliative care should be provided across all stages of dementia and that timely recognition of end of life remains a research priority to enable appropriate palliative care.^17^

Advanced dementia lasts an average of 2 years, but healthcare systems do not clearly recognise when someone with dementia reaches this stage.^18^ Characteristics of advanced dementia are underlined by the profound level of dependency on others to meet their basic needs, including progressive immobility, dysphagia and the limited ability to express their needs.^19^ This leaves people dying with dementia at a disadvantage, as research indicates that people with dementia are less likely to receive palliative care.^6,20^ Alternatively, people with dementia may reach the end of life before they progress to the advanced stages of dementia, where they may not receive end of life care and become hospitalised with potentially burdensome interventions until they die.^10^

This systematic review will explore how end of life is defined, and which methods of identifying end of life in dementia may be appropriate for future research and clinical practice.

## Aim and Objectives

This systematic review aimed to investigate how research studies have defined end of life in people living with dementia. The following research questions were identified:

1. How is end of life in people with dementia conceptualised and measured in research?
2. What are the factors that contribute to identifying end of life in people with dementia?
3. Does the setting of care for people with dementia influence how end of life is defined?

## Methods

A systematic review of quantitative and qualitative research studies following the Centre for Reviews and Dissemination guidance.^21^ This review followed the Preferred Reporting Items for Systematic Reviews and Meta-Analyses (PRISMA) as a reporting guideline.^22^ A protocol was registered with the PROSPERO database (CRD42020183968).

### Search strategy

Electronic databases MEDLINE, EMBASE, PsychInfo and CINAHL were searched from inception to April 2020. Search terms for dementia and end of life were used in combination with truncations and Boolean operators including AND and OR, to yield relevant results. The search terms used followed the guidance on using valid palliative care search terms in different databases, by Rietjens et al.^23^

An initial pilot search was conducted to refine the search strategy. Known key articles were identified within the search results, which confirmed good sensitivity. The full search applied to the MEDLINE database is outlined in Supplemental Table 1.

### Eligibility criteria

#### Inclusion criteria

Studies were included if they met the following criteria:

(1) Included adults of any age living with a dementia diagnosis. Family carers or healthcare professionals were included as they are likely to communicate or make decisions on behalf of the person dying with dementia.^24^
(2) Included a definition or specified criteria of end of life in dementia.
(3) Cohort studies, randomised controlled trials, qualitative studies and systematic reviews that referred to assessments or definitions for end of life in dementia, or were focused around palliative care in dementia.

#### Exclusion criteria

Studies were excluded if they:

(1) Did not specify palliative care or end of life.
(2) Were retrospective in design where people with dementia had already died, such as death register studies or biomedical studies, as these studies do not require identifying the dying phase.
(3) Were conference abstracts, theses, case studies, non-systematic literature reviews and editorial pieces.

### Study selection

All titles and abstracts for the papers retrieved from the search strategy were imported into EndNote X9 software, and de-duplicated. Article titles and abstracts were screened by one reviewer (BB) and 20% of all screened titles and abstracts were screened by other reviewers (ND, NK, KM).^25^ Discrepancies in the inclusion of studies were discussed, to reach agreement. Articles considered eligible at the title and abstract stage were screened using their full text against the eligibility criteria by one reviewer (BB). Thirty percent of the results of the full-text screening were then reviewed independently by three reviewers (ND, NK, KM). Any disagreement was discussed and a consensus of inclusion or exclusion of papers was made as a group.

### Quality appraisal

Eligible studies were assessed for methodological quality, using an adapted version of the Mixed Method Appraisal Tool.^26^ An adapted Mixed Method Appraisal Tool was used to meet the specific needs and research question of this review. The adapted quality appraisal tool was developed to help determine the quality of each study, and whether this may have contributed to how end of life was defined. Studies were considered high quality if they had clear eligibility criteria, minimal selection bias in sample recruitment, used appropriate data collection methods, and a definition of end of life in dementia. Studies were not excluded based on the results of the quality appraisal, however, they remained in the review for the discussion of research regarding end of life in dementia. Quality appraisal was completed by BB and checked by all other authors.

### Data extraction and analysis

Data extraction for included studies was completed by BB, using Microsoft Excel. Study details including the study design, aim, sample characteristics, eligibility criteria, end of life definition and the study setting were extracted from each paper. Data extraction was checked and discussed with all other authors.

A narrative synthesis,^27,28^ was conducted using tabulation and thematic analysis to synthesise studies identifying the key factors informing methods of defining end of life. We adopted an inductive approach, where one reviewer (BB) read the studies multiple times to facilitate thematic analysis. Patterns were identified by reviewing the methods, results and discussion sections. A colour coding system using highlighters on hard copy versions of papers, including potential themes were extracted. This was to ensure that the context of the themes was not lost.^28^ The initial themes were then refined into analytical themes. Regular meetings among all reviewers were arranged throughout, to discuss and finalise the findings. Any disagreement would have been resolved through consensus.

## Results

### Search results

**Figure 1.**
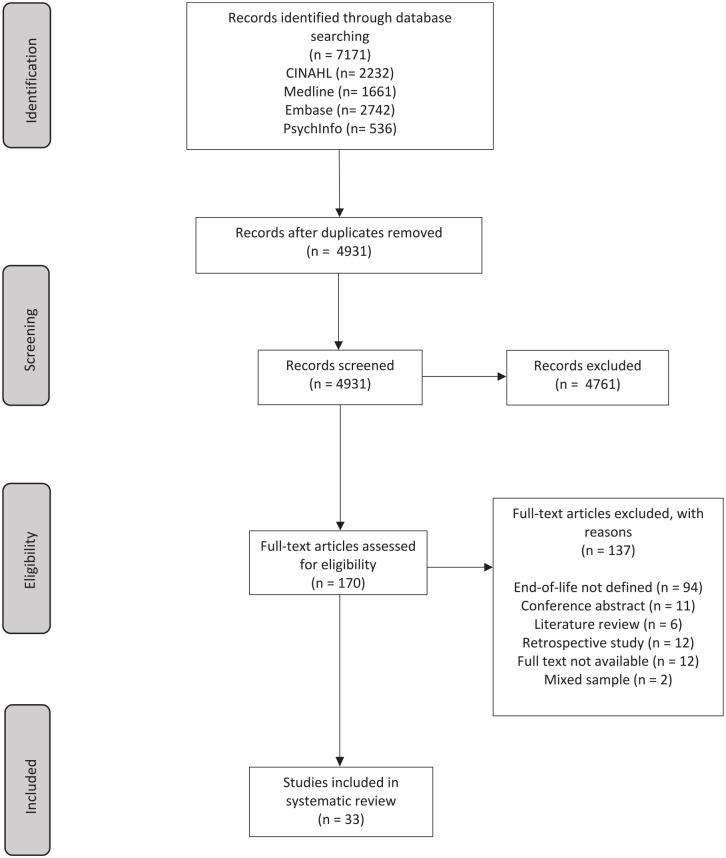
PRISMA flowchart of systematic search results.

The search strategy yielded 7171 results, of which 4931 studies were remaining after de-duplication. The full-text screening was completed for 170 studies, where 33 studies met the eligibility criteria for inclusion.^10,29–60^ Details of the search results and exclusion of studies can be found in Figure 1.

### Study characteristics

Table 1 provides the characteristics of the 33 studies. These studies included: 13 cohort,^32,35,36,38–40,44–46,49,52,53,56^ eight qualitative,^34,42,50,51,54,57,59,60^ five randomised controlled trials,^29,37,43,47,55^ and four evaluation designs.^30,31,33,48^ The remaining three studies used mixed-methods^10,41^ and quasi-experimental designs.^58^ Studies were undertaken in ten different countries: 15 studies in the United States of America (USA),^36–38,40–42,45–49,51–54^ five in the UK,^43,55,56,59,60^ four in Israel,^30–33^ two each in Italy^35,44^ and Australia,^29,34^ and one each in Switzerland,^39^ Germany,^57^ Netherlands,^10^ Canada^58^ and Japan.^50^

**Table 1.** Study characteristics (grouped by setting).

Authors	Country	Study design	Sample characteristics	Eligibility criteria	End of life definition	Care setting
Agar et al.^29^	Australia	Randomised controlled trial	286 nursing home residents with advanced dementia with family members and nursing home staff	Dementia diagnosis; Functional Assessment Screening Tool stage 6a or higher and stable for 1 month; Australia-Modified Karnofsky Performance Status score 50 or less	Functional Assessment Screening Tool stage 7c combined with functional dependency, predictive of average survival of less than 6 months	Nursing home
Andrews et al.^34^	Australia	Qualitative study	10 family members of advanced dementia residents in a Dementia Specific Unit	Family carers responsible for making health decisions for the person with advanced dementia; 18 years or older; person with advanced dementia to be a resident in the Dementia Specific Unit for at least 3 months	Global Deterioration Scale 6–7	Nursing home
Appollonio et al.^35^	Italy	Cohort study	103 female nursing home residents with different forms of dementia	Dementia diagnosis; nursing home admission of at least 3 months; Mini-Mental State Examination score of 18 or less; Clinical Dementia Rating score of 1 or more		Nursing home
Cadigan et al.^36^	USA	Cohort study	323 nursing home residents with advanced dementia	Any dementia diagnosis; Global Deterioration Scale of 7; aged 60 years or older; lived in nursing home for 30 days or longer; an appointed healthcare professional that could communicate in English		Nursing home
Cohen et al.^37^	USA	Randomised controlled trial	Intervention arm: 212 dyads. Control arm: 190 dyads. Dyads defined as healthcare proxies of nursing home residents with advanced dementia	Aged over 65 years; any dementia type; Global Deterioration Scale of 7; lived in nursing home over 90 days; had English speaking proxy		Nursing home
D’Agata and Mitchell^38^	USA	Cohort study	240 nursing home residents with advanced dementia	Aged 60 years or over; lived 30 days or longer; cognitive impairment due to dementia; Global Deterioration Scale of 7; an appointed healthcare proxy who could communicate in English	Cognitive Performance Scale of 5–6	Nursing home
Eicher et al.^39^	Switzerland	Cohort study	410 nursing home residents with advanced dementia	A dementia diagnosis; Cognitive Performance Scale of 5–6; informed consent by authorised representatives		Nursing home
Epstein-Lubow et al.^40^	USA	Cohort study	685,305 people with advanced dementia	Nursing home resident; enrolled in a Medicare fee-for-service plan; older 66 years; Cognitive Performance Scale of 5–6		Nursing home
Ernecoff et al.^41^	USA	Secondary data analysis	241 dyads of nursing home residents with late-stage dementia and their family decision-makers	Nursing home resident; 65 years old or older; dementia staged as 5–7 on Global Deterioration Scale; survived a 9-month follow up		Nursing home
Forbes et al.^42^	USA	Qualitative study	28 family members of nursing home residents with moderately severe to severe dementia	Family member of nursing home resident diagnosed with dementia, with a Cognitive Performance Scale of 4–6		Nursing home
Froggatt et al.^43^	UK	Randomised controlled trial	8 nursing homes (6 intervention and 2 control)	Nursing home residents: permanent resident in care home; lack mental capacity; Functional Assessment Screening Tool stage 6–7; has a key worker able to complete outcome tools		Nursing home
Di Giulio et al.^44^	Italy	Cohort study	482 nursing home residents with advanced dementia	Functional Assessment Screening Tool stage 7c or over; lived in nursing home for at least 6 months		Nursing home
Goldfeld et al.^45^	USA	Cohort study	323 nursing home residents with advanced dementia	Older than 60 years of age; any dementia diagnosis; Global Deterioration Scale of 7; available English speaking proxies to provide informed consent of their participation and the residents’ participation		Nursing home
Kiely et al.^48^	USA	Impact evaluation study	189 nursing home residents with advanced dementia and their healthcare proxies	Over 65 years old; Cognitive Performance Scale of 5–6; Global Deterioration Scale of 7; length of stay of 30 days or longer; availability of healthcare proxy willing to participate and communicate in English		Nursing home
Kiely et al.^49^	USA	Cohort study	323 nursing home residents with advanced dementia and their healthcare proxies	Age above 60 years; any dementia diagnosis; Global Deterioration Scale of 7; available English speaking proxies to provide informed consent		Nursing home
Kobayashi et al.^50^	Japan	Qualitative study	7 group home administrators (nurses or welfare care-workers)	Group home administrators required to have provided end of life care to residents with dementia in a group home	The period between the moment the group home administrators became aware of the signs of approaching death, and the actual death of the resident	Nursing home
Lopez et al.^51^	USA	Qualitative study	6 nursing home physicians and 14 nurses	Physicians and nurses from nursing homes that care for residents with advanced dementia aged 60 years and above; Global Deterioration Scale of 7		Nursing home
Reinhardt et al.^53^	USA	Cohort study	110 family members of nursing home residents with advanced dementia	Dementia diagnosis; Cognitive Performance Scale of 4–6; English or Spanish speaking; not receiving hospice care		Nursing home
Rosemond et al.^54^	USA	Qualitative study	16 family decision-makers of nursing home residents with advanced dementia who have died	English speaking family decision-makers of nursing home residents who had a dementia diagnosis; older than 65 years old; Global Deterioration Scale of 5–7		Nursing home
Schmidt et al.^57^	Germany	Qualitative study	30 nursing home residents with advanced dementia, 42 health professionals and 14 relatives	Residents: Over 65 years old; any dementia diagnosis; Global Deterioration Scale of 6–7; verbal inability to communicate. Health professionals: Directly involved in care of residents with advanced dementia; over 18 years old	Advanced dementia considered the final phase of life	Nursing home
Verreault et al.^58^	Canada	Quasi-experimental study	193 residents with advanced dementia and close family members (97 in intervention, 96 in usual care)	Terminal dementia; Functional Assessment Screening Tool stage 7e to 7f; profiles 13 and 14 on Functional Autonomy Measurement System	Terminal dementia; Functional Assessment Screening Tool stage 7e to 7f; scores 13–14 on Functional Autonomy Measurement System	Nursing home
Aminoff^30^	Israel	Outcome evaluation study	183 patients with advanced dementia admitted to a geriatric ward	Mini-Mental State Examination score 0/30; minimum Functional Independence Measure score 18/126; Functional Assessment Screening Tool stage 7c or higher	Aminoff Suffering Syndrome: high Mini-Mental State Examination score, less than 6-month survival for terminal patients, less than 1-month survival for dying patients, irreversible and intractable aggravation of medical conditions, suffering until deceased	Hospital
Aminoff and Adunsky^31^	Israel	Outcome evaluation study	71 patients with advanced dementia admitted to a geriatric ward	Mini-Mental State Examination score 0/30; minimum Functional Independence Measure score 18/126		Hospital
Aminoff and Adunsky^32^	Israel	Cohort study	134 end-stage dementia patients admitted to a long term geriatric ward	Mini-Mental State Examination score 0/30; minimum Functional Independence Measure score 18/126	Life expectancy of <6 months	Hospital
Aminoff et al.^33^	Israel	Impact evaluation study	103 bedridden patients with end-stage dementia	Mini-Mental State Examination score 0/30; minimum Functional Independence Measure score 18/126		Hospital
Hanson et al.^47^	USA	Randomised controlled trial	62 dyads of people with end-stage dementia and family decision-makers on admission to hospital	Patients: Aged 65 years or older; hospitalised with acute illness; dementia diagnosis; Global Deterioration Scale of 5–7; had eligible family decision-maker. Family decision-makers: Legally authorised representative for healthcare decisions; could communicate in English		Hospital
Ouchi et al.^52^	USA	Cohort study	51 patients aged over 70 with advanced dementia who visited the emergency department	Functional Assessment Screening Tool stage 7		Hospital
Sampson et al.^55^	UK	Randomised controlled trial	33 advanced dementia patient-carer dyads under emergency hospital admission (22 intervention arm, 11 control arm)	Functional Assessment Screening Tool stage 6d or above; unplanned admission for a treatable acute medical illness; have an informal carer who can give informed consent for patients		Hospital
Hanrahan et al.^46^	USA	Cohort study	45 patients with severe dementia	National Hospice Organisation guidelines: Functional Assessment Screening Tool score 7c or above; dependence for all activities of daily living; severe comorbidities related to advanced dementia (i.e. pneumonia, sepsis); unable to maintain sufficient fluid and calorie intake to sustain life	Functional Assessment Screening Tool stage of 7c	Hospice
Yeh et al.^59^	UK	Qualitative study	29 homecare workers and 13 homecare managers who cared for people with dementia	Homecare agency: Providing care for older people in their home, including those with dementia. Homecare workers: To understand English to participate	Last years of life	Home
Sampson et al.^56^	UK	Cohort study	85 people with advanced dementia in nursing homes or their own homes	Dementia diagnosis; over 65 years old; Functional Assessment Screening Tool stage 6e or above		Home and nursing home
Van der Steen et al.^10^	Netherlands	Mixed-method study	10 caregivers of people with advanced dementia Experts involved in terminal care services in dementia	Caregivers of spouses with advanced dementia from the support group at day centre where spouses previously attended	Last days or weeks or at most, a few months of life	Home and nursing home
Sampson et al.^60^	UK	Qualitative study	20 people with dementia in care homes and hospitals; and 22 staff members involved in dementia care	Functional Assessment Screening Tool stage 6c or higher		Hospital and nursing home

### Quality appraisal

All included studies were rated as good quality using the modified quality appraisal. The quality of two cohort studies^35,52^ was at risk of bias due to using convenience sampling in a single nursing home, which resulted in sampling bias due to a possible lack of a representative sample.^61^ The mixed-methods study conducted by Van der Steen et al.^10^ demonstrated low quality within the eligibility criteria, as it cannot be replicated accurately in regards to determining the dementia severity of participants. The full quality appraisal is provided in Supplemental Table 2.

### End of life definitions

Eleven studies had explicit definitions of end of life in dementia.^10,29,30,32,34,38,46,50,57–59^ Four studies used cut-off scores from validated tools^34,38,46,58^ (see Table 1). One study used a prognosis of less than 6 months survival,^32^ which was derived from the National Hospice Organization eligibility guidelines for people with dementia.^62^ Four studies used a range of descriptions,^10,50,57,59^ and two studies used a combination of cut-off scores from validated tools and descriptions.^29,30^ There was a consistent pattern of cut-off scores from validated tools used to define end of life, where stages 5–6 of the Cognitive Performance Scale^63^ were commonly used throughout the studies. These scores referred to severe cognitive impairment, where the person had severe dementia, and total dependency in activities of daily living.^63,64^

In contrast, descriptive definitions were broad and displayed ambiguity in what was defined as end of life in dementia. Some descriptions contradicted each other; where Van der Steen et al.^10^ described end of life as a person with dementia being in their last days, weeks or months of life, and Schmidt et al.^57^ referred to advanced dementia as being the final phase of life, which can persist over several years. The remaining 22 studies did not provide an explicit definition of end of life in dementia. Where definitions were not provided within the studies, we used the inclusion criteria for participant recruitment that were used to establish how end of life in dementia was defined.

### Measures

Eight different validated tools^63–70^ were used within the studies’ eligibility criteria in 30 out of 33 studies, to identify people living with dementia approaching the end of life^29–49,51–58,60^ (Table 2). Nine studies were identified as using 2–3 different validated scales interchangeably within the same study, where cut-off scores of 6 and above on the Global Deterioration Scale^65^; 5–6 on the Cognitive Performance Scale^63^; 0/30 on the Mini-Mental State Examination^66^; and 18/126 on the Functional Independence Measure,^67^ were classed as end of life.^29–33,35,38,48,57^ The Global Deterioration Scale,^65^ which assesses cognition, was the most commonly used validated tool applied in 12 studies, where the cut-off scores referred to moderate dementia (Global Deterioration Scale 5) to severe dementia (Global Deterioration Scale 7).^34,36–38,41,45,47–49,51,54,57^ The Functional Assessment Screening Tool (64), which focuses on function, was the second most commonly used tool to define end of life in dementia, where Table 2 shows six different stages of the Functional Assessment Screening Tool applied in ten studies.^29,30,43,44,46,52,55,56,58,60^ The Functional Assessment Screening Tool stages ranging from 6a (requires physical assistance with clothing) to 7f (inability to hold head up),^64^ were used to identify someone at the end of life with dementia throughout the studies.

**Table 2.** Validated tools used to define end of life in dementia.

	Global Deterioration Scale^65^	Cognitive Performance Scale^63^	Functional Assessment Screening Tool^64^	Mini-Mental State Examination^66^	Functional Independence Measure^67^	Clinical Dementia Rating^68^	Australia-Modified Karnofsky Performance Status^69^	Functional Autonomy Measurement System^70^
Agar et al.^29^			6a or above				50 or less	
Aminoff^30^			7c or above	0/30	Minimum score 18/126			
Aminoff and Adunsky^31^				0/30	Minimum score 18/126			
Aminoff and Adunsky^32^				0/30	Minimum score 18/126			
Aminoff et al.^33^				0/30	Minimum score 18/126			
Andrews et al.^34^	6 to 7							
Appollonio et al.^35^				18 or less		1 or above		
Cadigan et al.^36^	7							
Cohen et al.^37^	7							
D’Agata and Mitchell^38^	7	5 to 6						
Eicher et al.^39^		5 to 6						
Epstein-Lubow et al.^40^		5 to 6						
Ernecoff et al.^41^	5 to 7							
Forbes et al.^42^		4 to 6						
**Froggatt et al.** ^43^			6 to 7					
Di Giulio et al.^44^			7c or above					
Goldfeld et al.^45^	7							
Hanrahan et al.^46^			7c or above					
Hanson et al.^47^	5 to 7							
Kiely et al.^48^	7	5 to 6						
Kiely et al.^49^	7							
Lopez et al.^51^	7							
Ouchi et al.^52^			7					
Reinhardt et al.^53^		4 to 6						
Rosemond et al.^54^	5 to 7							
**Sampson et al.** ^55^			6d or above					
Sampson et al.^56^			6e or above					
Sampson et al.^60^			6c or above					
Schmidt et al.^57^	6 to 7							
**Verreault et al.** ^58^			7e to 7f					13 and 14

Overall, the cut-off scores used in the studies required considerable decline in cognition and function to classify end of life in dementia (Table 2). The characteristics of each validated tool and the main domains used to assess dementia severity, are outlined in Supplementary Table 3.

### Care setting

Four care settings for people living with dementia were identified from the 33 studies (Table 1). Most studies were conducted in the nursing home setting (21 studies),^29,34–45,48–51,53,54,57,58^ followed by the hospital setting (7 studies).^30–33,47,52,55^ Hospice and home settings were the focus only in one study each.^46,59^ Two studies were conducted in a combination of care settings, where one study was in home and nursing home settings,^10^ and another included hospital and nursing home settings.^56^ The care setting did not influence how end of life in dementia was defined. There was a variety of definitions used in the nursing home setting to establish end of life in dementia in the USA.^36–38,40–42,45,48,49,51,53,54^ However, one hospice-based study in the USA^46^ highlighted that the Functional Assessment Screening Tool^64^ assumes a strict stage-by-stage progression, which is a requirement to determine end of life within the National Hospice Organization guidelines.^62^ However, people with dementia may not progress through such stages in the predetermined order outlined by the tool. Therefore, the characteristics of the Functional Assessment Screening Tool^64^ fail to recognise the variability of dementia progression.

Overall, the definitions used to define end of life in dementia did not differ between the care settings or countries, and there was no consistency of the measures used to establish end of life in specific care settings.

### Themes identified

Narrative synthesis using tabulation and thematic analysis identified three main themes as crucial components in how end of life was defined in dementia. The themes included limitations in existing measures to define end of life in dementia, family knowledge and staff knowledge. These core themes were interlinked, as one theme influenced the occurrence of another theme, such as family and staff knowledge.

### Limitations in existing measures to define end of life in dementia

Six studies^30–33,35,46^ advocated for alternative measures to identify end of life, as the current measures based on cognition and ambulatory function were reported to be ineffective in identifying end of life in dementia. Aminoff and Adunsky^32^ recommended assessing the level of suffering in people dying with dementia. The authors^32^ referred to suffering as a state of psychological distress, spiritual concerns and various presentations of physical pain. They assessed suffering of terminal dementia patients over time, from admission to a geriatric ward to the last day of life, and found 63% of people with advanced dementia died with high levels of suffering and had shorter survival times.^31^ Following these findings, the authors developed the Aminoff Suffering Syndrome tool as a potential method to identify people at the end of life with dementia. In a later evaluation study, the authors argued that the tool was sensitive to detecting when someone living with dementia might be at the end of life^30^ (Table 1).

Hanrahan et al.^46^ in their assessment of the characteristics of people living with dementia eligible for hospice admission, found that almost half of their study sample presented stages of the Functional Assessment Screening Tool in a non-sequential order. Therefore, these patients were not deemed to be eligible for hospice care, according to the National Hospice Organization guidelines.^62^ Forty-four percent of individuals with dementia presented mobility problems that were characteristic of the Functional Assessment Screening Tool stage 7c. However, these patients did not present features of earlier stages of the tool, such as verbal inability (stage 7a), as they were still able to speak in sentences. Therefore, these participants were not classified as Functional Assessment Screening Tool stage 7a or higher.^46^ Additionally, these participants continued to have potentially burdensome care such as antibiotic therapy, which did not prolong their lives.^46^ Therefore, these results demonstrated the limitation of using specific scale-based measures to identify end of life within this population.

### Family knowledge

Family carers had limited understanding that people living with dementia can die from dementia. Most relatives did not consider dementia to be the cause of their relative’s decline and considered death to be unrelated to dementia.^34^ Family carers were not aware of the dying phase of dementia and believed that their relatives would die from a ‘big event’ such as a stroke, instead of dementia itself. One family carer expressed:

‘I don’t think she [Mum] will die from dementia, I think she will die from a heart attack or stroke . . . some other medical condition but not dementia . . . do people die from dementia? I’ve never . . . heard of people dying [from it]’.^34^

Family carers’ wishes of their relatives having a natural death were contradicted with their additional wishes to continue potentially burdensome treatments. Treatments included antibiotic therapy and respiratory ventilation, as they did not view conditions such as pneumonia to be part of the natural death of someone dying with dementia.^42^

### Staff knowledge

Limited knowledge of end of life in dementia among healthcare professionals was also identified within the studies. In the nursing home setting, there was no systematic training of end of life and dementia care, which resulted in staff using their previous experiences to estimate when a person with dementia was at the end of life.^10,50^ Nurses employed in nursing homes in some counties had minimal qualifications, mostly under university degree level, who were supported by unqualified nurse assistants, with no previous training or supporting policies in end of life in dementia.^10^ This lack of knowledge may have been the result of the type of reimbursement policies enforced by nursing homes, as some policies did not reimburse nursing homes for initiating palliative care.^45^ For example, nursing homes in the USA were primarily reimbursed by the Medicaid fee-for-service policy, which did not reimburse nursing homes for preventing unnecessary hospital transfers.^45^ Alternatively, Medicare managed-care plans prevented hospital transfers by employing on-site nurse practitioners, who were specialised in providing palliative care and end of life care in dementia.^45^ This service was underused in people with dementia in nursing homes due to increased costs for beneficiaries.^45^ Therefore, the care plans provided in such nursing homes did not include planning for end of life in dementia.

In the emergency department care setting, doctors lacked the knowledge and understanding of the importance of palliative care consultation for people approaching end of life with dementia. In a study examining the rate of emergency department initiated palliative care consultation requests for people with dementia in the USA, 68% of cases did not have palliative care consultations due to doctors’ lack of knowledge within this speciality.^52^

## Discussion

Defining end of life in dementia is complex due to the potentially long and unpredictable trajectory of dementia. Many people with dementia may never reach the advanced stages and may die from other causes earlier in the trajectory.^71^ This paper is the first to provide a systematic review of how end of life is measured and defined in dementia research. Our findings highlight the inconsistency across studies in how end of life is defined. Studies fail to address the complexity of defining end of life, tending to use a single-domain validated tool to capture populations who may be nearing end of life. Some studies focused on cognitive impairment and others on functional decline, however all neglected to consider the holistic needs of the individual.

Families did not consider dementia to be a terminal condition, and thus had limited understanding of how their relatives should be cared towards the end of life.^34,42^ Similarly, many staff did not have the training and experience of end of life care and dementia. Both staff and families were faced with uncertainty about estimating if a person was at the end of their life. Given the complexity of defining end of life and the unpredictable trajectory, focusing on the needs of the individual may be more suitable than focusing on defining end of life.

### Appropriateness of end-of-life measures

Multiple validated tools were used interchangeably with cut-off scores indicating advanced dementia were mostly used to establish or define end of life in dementia.^29–33,35,38,48,57^ Aminoff^30^ defined end of life in dementia by combining the Functional Assessment Screening Tool stage 7c and the Mini-Mental State Examination score 0/30. However, the Mini-Mental State Examination shows a floor effect when it assesses dementia at the advanced stages. Additionally, the Mini-Mental State Examination^66^ is not a dementia staging tool, but rather a tool to measure cognitive impairment for all causes, not only dementia.^63^ Therefore, the Mini-Mental State Examination^66^ may not be appropriate for assessing end of life in dementia, as it generalises all presentations of severe dementia into a zero score, whilst the Functional Assessment Screening Tool categorises end of life in advanced dementia into five sub-stages (stages 7a to 7e).^72^ However, the Functional Assessment Screening Tool^64^ is not without its limitations, as it assumes a sequential pattern of deterioration in people with dementia.^73,74^ This was shown in the cohort study conducted by Hanrahan et al.,^46^ where almost half of the study sample were not eligible for hospice admission, because their dementia did not progress in this ordinal way. Therefore, this scale may also be inappropriate to use for people approaching end of life with dementia as there is great heterogeneity in how people progress through different stages of functional decline.^75^

Four studies stated a 6-month prognosis to establish end of life in dementia,^29,30,32,44^ however their findings suggested this prognosis to be an inappropriate indicator of end of life in dementia. For example, in a study by Aminoff and Adunsky,^32^ 47% of participants survived longer than 6 months and only 46% of participants with advanced dementia died during an 18-month study by Agar et al.^29^ Such findings in this review are similar with other studies, where research by Sampson et al.^56^ showed that only one third of participants with advanced dementia died by the end of the 9-month prospective study.

### Implications for research, policy and clinical practice

This systematic review raises awareness of the gap in knowledge of end-of-life identification in research and clinical practice. Validated scales including the Global Deterioration Scale and the Clinicians Global Impression of Change, are used to determine dementia severity in research.^76^ However, our review demonstrates there is a lack of evidence supporting the use of these scales in clinical practice for end of life in dementia. The Clinical Frailty Scale^77^ is used in the UK within the National Health Service to assess the risk of mortality among older adults, who are admitted into hospital care.^78^ Although the Clinical Frailty Scale has shown a strong correlation between dementia and frailty, there is a lack of evidence on the effectiveness of the scale in identifying end of life in dementia.^79^

Quality of life scales for people with dementia are also used to determine palliative care needs of this population, such as the Alzheimer’s Disease-related Quality of Life Scale.^76^ The Alzheimer’s Disease-related Quality of Life Scale assesses quality of life across all stages of dementia severity, and is used widely within the American healthcare system.^80^ However, this scale was created for self-completion of people with dementia, which may not be possible in people with severe cognitive impairment.^81^

Symptoms of end of life in dementia including pain, apathy and dysphagia, may require specialist skills based on individual need.^82^ Other symptoms include psychosocial aspects such as depression, anxiety and irritability, which tend to be overlooked in contrast to the physical needs of people at the end of life with dementia.^83^ Acknowledging the psychosocial needs of caregivers is also important for enabling a holistic approach towards end of life in dementia, as the deterioration of the recipient’s health induces burden and emotions including sadness and anger, which can affect their ability to provide adequate care.^84^ Palliative care staff have reported their limited capacity and resources to provide specialist palliative care for people with dementia,^85^ but may play an important role in supporting generalist staff in addressing palliative care needs. The need for a multidisciplinary and palliative approach tailored for people living with dementia encompassing physical, medical and psychosocial needs is required.

Future research should question the value in using validated scales to define end of life, and the use of stages to define end of life. Other studies have selected their sample based on clinical opinion and family views of whether their relative is at the end of life,^86–88^ however this is a subjective approach to inclusion. Future research should consider the development of a consensus statement on end of life in dementia.

The inconsistency and complexity of defining end-of-life care identified in this review, suggests there is a need to refocus our discussion from defining end of life based on stage of disease, and consider end of life beyond prognostication, responding to individual needs to improve end-of-life care in dementia. Refocussing this attention clinically would encourage clinicians to manage and work with uncertainty and consider a needs-based approach for their patients.

### Strengths and limitations

This review has several strengths including the systematic approach to identify relevant studies, following guidance from the Centre for Review and Dissemination^21^ and guidance on narrative synthesis,^27^ and registering the protocol PROSPERO to ensure transparency throughout the review.^89^ Key relevant papers were identified prior to the search to ensure the search strategy identified these key papers as indication of the specificity and sensitivity of the search. The quality appraisal tool was adapted to meet the specific requirements of this review, as there were no validated tools previously developed concerning the research question. However, the tool was not a validated measure of methodological quality.

The review findings are limited by the majority of the participants with dementia being predominantly female and white, and most studies in this review were conducted in high income countries, thus not representing dementia in low and middle income countries. Countries with higher populations of people with dementia including Japan^90^ had substantially fewer publications in this review, compared to the USA, where most of the literature originated from. Therefore, more research is required focusing on other countries with rapidly ageing populations and growing numbers of people living with dementia.

## Conclusion

This systematic review presents evidence that a definition for end of life in dementia remains poorly defined, and unrepresentative of the general population with dementia. Research investigating palliative care that only includes cognitive or functional decline, may fail to recognise other significant signs and unmet needs relevant to dementia and end of life. We suggest that researchers and healthcare professionals in dementia care accept the complex nature of end of life in dementia between and within individuals. We advocate for a transition beyond defining end of life by disease-stage, and to consider signs beyond cognitive and functional decline. Identifying the appropriate signs and needs of individuals at the end of life with dementia will require further research, but this will be imperative to an improved understanding of end of life in dementia. This approach may provide an improved response to end-of-life care for people with dementia and their families.

## Supplemental Material

sj-pdf-1-pmj-10.1177_02692163211025457 – Supplemental material for Defining end of life in dementia: A systematic review Click here for additional data file. Supplemental material, sj-pdf-1-pmj-10.1177_02692163211025457 for Defining end of life in dementia: A systematic review by Bria Browne, Nuriye Kupeli, Kirsten J Moore, Elizabeth L Sampson and Nathan Davies in Palliative Medicine
