# Supplementary material for: Defining end of life in dementia: A systematic review
Source: Palliat Med. 2021 Jun 17;35(10):1733–46. doi: 10.1177/02692163211025457 (PMC8637358; doi:10.1177/02692163211025457)
Supplement: sj-pdf-1-pmj-10.1177_02692163211025457 – Supplemental material for Defining end of life in dementia: A systematic review [file sj-pdf-1-pmj-10.1177_02692163211025457.pdf]

Supplementary Materials

Table 1 - Full search for databases

#	**Search term**
1.	Dementia OR Dementia, Vascular
2.	Alzheimer Disease
3.	Palliative Care
4.	Terminal Care
5.	(((end adj3 life) OR palliat* OR (advanced or late)) adj3 stage*
6.	3 OR 4 OR 5
7.	(Dementia OR Alzheimer*)
8.	1 OR 2 OR 7
9.	6 AND 8
10.	Limit 9 to (English language AND humans AND all adult)

Table 2 - Quality appraisal of studies

	Is the research design appropriate to answer the research question?	Clear eligibility criteria?	Appropriate data collection measures used?	Are participants recruited in a way that minimises selection bias?	Definition of end of life provided?
**Agar et al. (2017)(34)**	Y	Y	Y	Y	Y
**Aminoff (2014)(35)**	Y	Y	Y	Y	Y
**Aminoff & Adunsky (2004)(36)**	Y	Y	Y	Y	N
**Aminoff & Adunsky (2006)(37)**	Y	Y	Y	Y	N
**Aminoff et al. (2004)(38)**	Y	Y	Y	Y	N
**Andrews et al. (2017)(39)**	Y	Y	Y	Y	Y
**Appollonio et al. (2005)(40)**	Y	Y	Y	N	N
**Cadigan et al. (2012)(41)**	Y	Y	Y	Y	N
**Cohen et al. (2019)(42)**	Y	Y	Y	Y	N
**D'Agata & Mitchell (2008)(43)**	Y	Y	Y	Y	Y
**Eicher et al. (2016)(44)**	Y	Y	Y	Y	N
**Epstein-Lubow et al. (2015)(45)**	Y	Y	Y	Y	N

	Is the research design appropriate to answer the research question?	Clear eligibility criteria?	Appropriate data collection measures used?	Are participants recruited in a way that minimises selection bias?	Definition of end of life provided?
**Ernecoff et al. (2019)(46)**	Y	Y	Y	Y	N
**Forbes et al. (2000)(47)**	Y	Y	Y	Y	N
**Froggatt et al. (2018)(48)**	Y	Y	Y	Y	N
**Di Giulio et al. (2019)(49)**	Y	Y	Y	Y	N
**Goldfeld et al. (2013)(50)**	Y	Y	Y	Y	N
**Hanrahan et al. (1999)(51)**	Y	Y	Y	Y	Y
**Hanson et al. (2019)(52)**	Y	Y	Y	Y	N
**Kiely et al. (2006)(53)**	Y	Y	Y	Y	N
**Kiely et al. (2012)(54)**	Y	Y	Y	Y	N
**Kobayashi et al. (2008)(55)**	Y	Y	Y	Y	Y
**Lopez et al. (2017)(56)**	Y	Y	Y	Y	N
**Ouchi et al. (2014)(57)**	Y	Y	Y	N	N
**Reinhardt et al. (2015)(58)**	Y	Y	Y	Y	N
**Rosemond et al. (2017)(59)**	Y	Y	Y	Y	N

	**Is the research design appropriate to answer the research question?**	**Clear eligibility criteria?**	**Appropriate data collection measures used?**	**Are participants recruited in a way that minimises selection bias?**	**Definition of end of life provided?**
**Sampson et al. (2011)(60)**	Y	Y	Y	Y	N
**Sampson et al. (2018)(61)**	Y	Y	Y	Y	N
**Sampson et al. (2019)(65)**	Y	Y	Y	Y	N
**Schmidt et al. (2018)(62)**	Y	Y	Y	Y	Y
**Van der Steen et al. (2017)(9)**	Y	N	Y	Y	N
**Verreault et al. (2018)(63)**	Y	Y	Y	Y	Y
**Yeh et al. (2019)(64)**	Y	Y	Y	Y	Y

Table 3 -Descriptions of validated tools

Tool	Description	Scoring Range
**Australia-Modified Karnofsky Performance Status (AKPS)(68)**	Observation to assess tasks related to activity, work and self-care	Score 10-100<br>Single score in increments of 10<br>(decreasing score indicates reduced performance status)
**Clinical Dementia Rating (CDR)(67)**	Semi-structured interview with patient and appropriate proxy to assess cognition	Score 0-3<br>5-point rating scale<br>(increasing score indicates increased dementia severity)
**Cognitive Performance Scale (CPS)(62)**	Semi-structured interview and observation with patient and appropriate proxy to assess cognition	Score 0-7<br>7-point rating scale<br>(increasing score indicates increased cognitive impairment)
**Functional Assessment Screening Tool (FAST)(63)**	Observation to assess functional capacity in dementia	Stages 1-7<br>11 substages 6a-6e, 7a-7f<br>(increasing stages indicates increased dementia severity)
**Functional Autonomy Measurement System (SMAF)(69)**	Semi-quantitative measure to assess disability and handicap, to guide care service needs	Levels 0-4<br>29 items assessed<br>(increasing level for items indicates increased dependence)
**Functional Independence Measure (FIM)(66)**	Observation to assess the level of function and cognition	Score 1-7 per item<br>18 items assessed<br>(decreasing score indicates increased dependence)
**Global Deterioration Scale (GDS)(64)**	Observation to assess cognitive function in dementia	Stages 1-7<br>(increasing score indicates increased dementia severity)
**Mini-Mental State Examination (MMSE)(65)**	Structured 30-item questionnaire to assess cognitive function	30 points<br>(decreasing score indicates increased cognitive impairment)
